# N6-methyladenosine reader YTHDF family in biological processes: Structures, roles, and mechanisms

**DOI:** 10.3389/fimmu.2023.1162607

**Published:** 2023-03-14

**Authors:** Lin Chen, Yang Gao, Simiao Xu, Jinxiong Yuan, Min Wang, Tianyu Li, Jun Gong

**Affiliations:** ^1^ Department of Biliary-Pancreatic Surgery, Affiliated Tongji Hospital, Tongji Medical College, Huazhong University of Science and Technology, Wuhan, China; ^2^ Division of Endocrinology, Affiliated Tongji Hospital, Tongji Medical College, Huazhong University of Science and Technology, Branch of National Clinical Research Center for Metabolic Disease, Wuhan, China; ^3^ Trauma Center/Department of Emergency and Traumatic Surgery, Affiliated Tongji Hospital, Tongji Medical College, Huazhong University of Science and Technology, Wuhan, China

**Keywords:** M6A, YTHDF, biological process, cancer, clinical applications

## Abstract

As the most abundant and conserved internal modification in eukaryote RNAs, N6-methyladenosine (m^6^A) is involved in a wide range of physiological and pathological processes. The YT521-B homology (YTH) domain-containing family proteins (YTHDFs), including YTHDF1, YTHDF2, and YTHDF3, are a class of cytoplasmic m^6^A-binding proteins defined by the vertebrate YTH domain, and exert extensive functions in regulating RNA destiny. Distinct expression patterns of the YTHDF family in specific cell types or developmental stages result in prominent differences in multiple biological processes, such as embryonic development, stem cell fate, fat metabolism, neuromodulation, cardiovascular effect, infection, immunity, and tumorigenesis. The YTHDF family mediates tumor proliferation, metastasis, metabolism, drug resistance, and immunity, and possesses the potential of predictive and therapeutic biomarkers. Here, we mainly summary the structures, roles, and mechanisms of the YTHDF family in physiological and pathological processes, especially in multiple cancers, as well as their current limitations and future considerations. This will provide novel angles for deciphering m^6^A regulation in a biological system.

## 1 Introduction

In recent years, more than 170 different chemical RNA modifications have been identified, drawing more attention to the epitranscriptome (1). Among them, N6-methyladenosine (m^6^A), which adds a methyl group to the sixth nitrogen atom of adenine, is the most abundant internal transcriptome modification in eukaryotes (2, 3). By identifying the consensus motif “RRACH” (R = A/G; H = A/C/U), m^6^A usually occurs in the 3’ untranslated region (3’UTR) and coding sequence (CDS), especially in the vicinity of stop codons (4, 5). Accordingly, m^6^A modification regulates the metabolism of multiple types of RNAs and are ultimately participating in various pathophysiological processes.

The m^6^A methylation is dynamic and reversible, regulated by a series of m^6^A-modifying enzymes which can be classified into “writers”, methyltransferases that install m^6^A modifications, and “erasers”, demethylases that remove m^6^A from mRNA, as well as “readers” that recognize and bind to m^6^A-modified mRNA to mediate their ultimate fate. Methyltransferase complex (MTC) is the main “writers”, including methyltransferase like 3/14 (METTL3/14), Wilms’ tumor 1-associating protein (WTAP) (6, 7). They catalyze the formation of m^6^A methylation synergistically. Conversely, the fat mass and obesity-associated protein (FTO) and AlkB homolog 3/5 (ALKBH3/5) that belong to the “erasers” act as key proteins in m^6^A demethylation (8, 9). Moreover, “readers” are important m^6^A binding proteins such as YTHDFs, YTH domain-containing 1/2 (YTHDC1/2), heterogeneous nuclear ribonucleoproteins (HNRNP) family, insulin-like growth factor 2 mRNA-binding proteins (IGF2BP1/2/3), and eukaryotic initiation factor 3 (eIF3) (5, 10–16). They influence RNA splicing, export, translation, and decay, and then regulate diverse downstream signaling pathways.

The YTHDF family is the most studied “readers” of m^6^A, which includes YTHDF1, YTHDF2, and YTHDF3. They regulate the translation and stability of target mRNAs to alter the expression of downstream molecules, thus affecting diverse biological processes (10, 17). In this review, we summarize the structures and functions of the YTHDF family, especially the m^6^A-binding specificity. Moreover, we focus on its underline mechanisms in multiple physiological and pathological processes, especially in tumors, hoping to provide possible application value.

## 2 M^6^A methylation regulators

In “writers”, MTC is the main component that catalyzes the formation of m^6^A. Among them, METTL3 installs methyl groups in S-adenosylmethionine to RNA target sites, while METTL14 selects RNA adenine bases and stabilizes the catalytic process (6, 18, 19). WTAP, RBM15/15B, VIRMA, and ZC3H13 are also components of the MTC, directing complexes to nuclear speckles as well as RNA sites (7, 20–22). In addition to MTC, METTL16, ZCCHC4, and METTL5 also can catalyze m^6^A modification of specific RNAs (23–25). In contrast, FTO and ALKBH3/5 act as key “erasers” proteins in m^6^A demethylation (8, 9, 26). FTO and ALKBH5 target mRNA and are associated with obesity and spermatogenesis, respectively (9, 27). Whereas ALKBH3 removes m^6^A on tRNA (26).

Moreover, “readers” are required in m^6^A-regulated diverse downstream signaling pathways. For example, YTHDC1 promotes mRNA splicing in the nucleus as well as nuclear export (11, 12). Furthermore, YTHDC1 accelerates the function of XIST to silence the transcription of genes on the X chromosome (20). Interestingly, YTHDC2 promotes mRNA translation with a concomitant decrease in mRNA abundance and has ATPase and 3’ to 5’ RNA helicase activities (13, 28). In addition, the HNRNP family regulates the alternative splicing of mRNA through an “m^6^A-switch” mechanism (29–33). IGF2BPs stabilize target mRNAs in different ways under normal and stress conditions (15). And eIF3 binds m^6^A on the 5’UTR of mRNA and promotes mRNA translation in a cap-independent manner (16).

**Figure 1:**
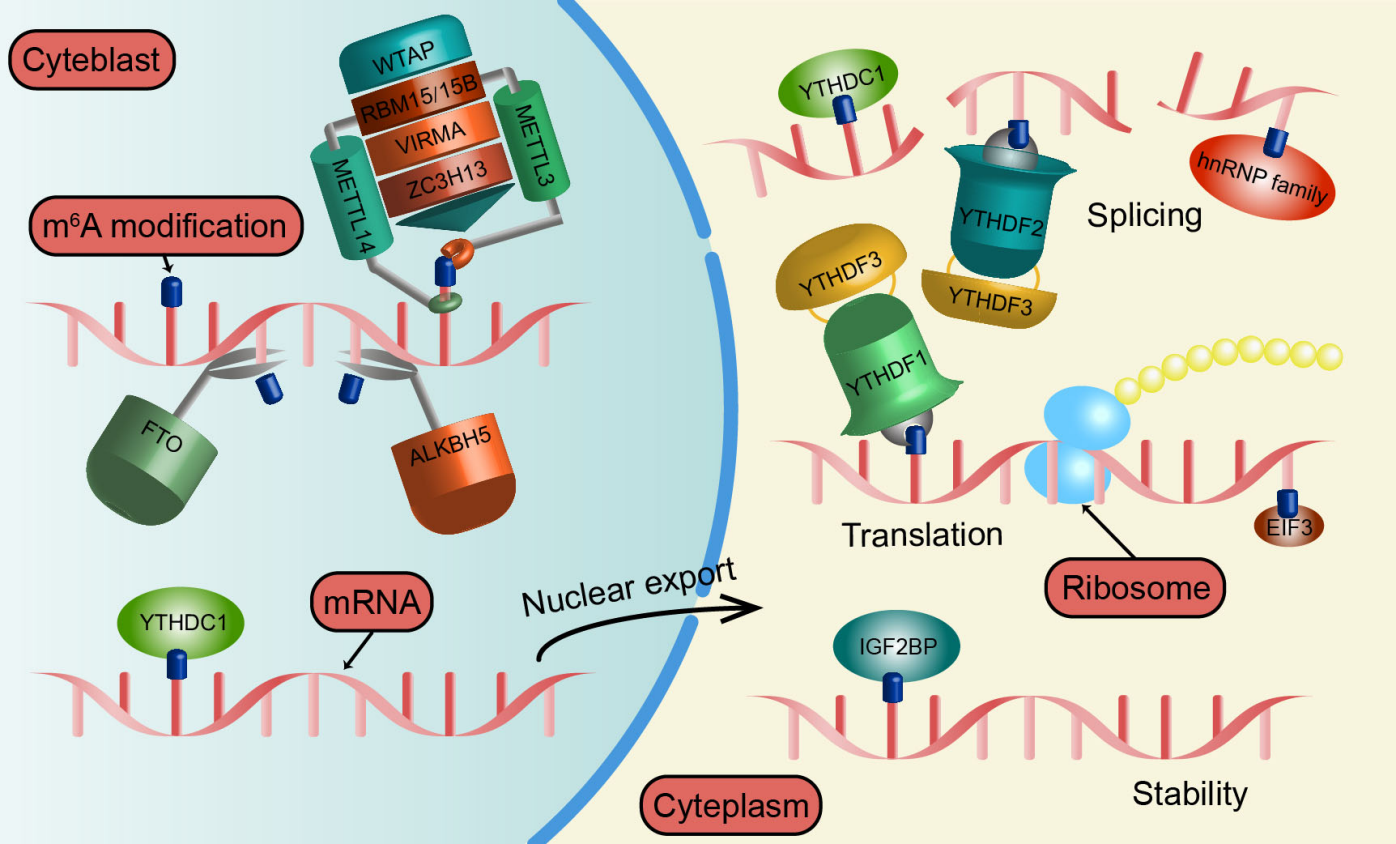
The regulation mechanism of m6A modification. METTL3, METTL14, WTAP, RBM15, VIRMA, and ZC3H13 all belong to the “writers” and catalyze the formation of m6A modification by constituting MTC. The “erasers” includes FTO and ALKBH5, which act as key proteins in m6A demethylation. YTHDF1/2/3, YTHDC1, IGF2BP, hnRNP family, and EIF3 as “readers” that bind to m6A and affect RNA splicing, output, translation, and decay.

The YTHDF family was identified by selecting proteins containing the YTH domain and subsequently obtained in pull-down experiments using methylated RNA bait (5, 34, 35). Now, the features of the YTHDF family have been gradually unraveled. The YTH domains of YTHDFs have a hydrophobic pocket, which is critical to the recognition of m^6^A in the cytoplasm (36). But the role of each protein is different, for example, YTHDF1 promotes RNA translation, YTHDF2 facilitates RNA decay, and YTHDF3 exhibits a dual function depending on its binding partner (37). Thus, the YTHDF family is closely associated with many cancers and other biological processes (**Figure 1**).

## 3 The structures and functions of the YTHDF family

The YTHDF family is composed of a C-terminal YTH domain and an N-terminal domain rich in P/Q/N (Pro/Gln/Asn). The YTH domain is the basis of recognizing m^6^A RNA specifically and its targeted position and consensus sequence are similar to the distribution pattern of m^6^A sites on mRNA (20, 38). YTH domain can also directly bind to N1-methyladenosine (m^1^A), but with a lower affinity than m^6^A (39). The prion-like low-complexity sequence regions (LCRs) of the N-terminal domain are associated with the liquid-liquid phase separation (LLPS) (40). The mRNA-YTHDF complexes are located in different membrane-less compartments in the cytoplasm, such as processing bodies (P-bodies), stress granules (SGs), or neuronal granules, which are the result of LLPS and can be enhanced by multivalent m^6^A modifications (41). Proteomic studies revealed that YTHDFs can be phosphorylated and myristoylated to regulate their expression and clustering (42). Additionally, the EGFR/SRC/ERK pathway stabilizes YTHDF2 protein by phosphorylating YTHDF2 at serine39 and threonine381 in glioblastoma cells (43). YTHDF2 can also be SUMOylated at site K571, thereby enhancing its binding affinity with m^6^A-modified mRNAs and accelerating cancer advancement (44). Therefore, targeting post-translational modifications represent a novel opportunity for YTHDFs to regulate their functions.

**Figure 2:**
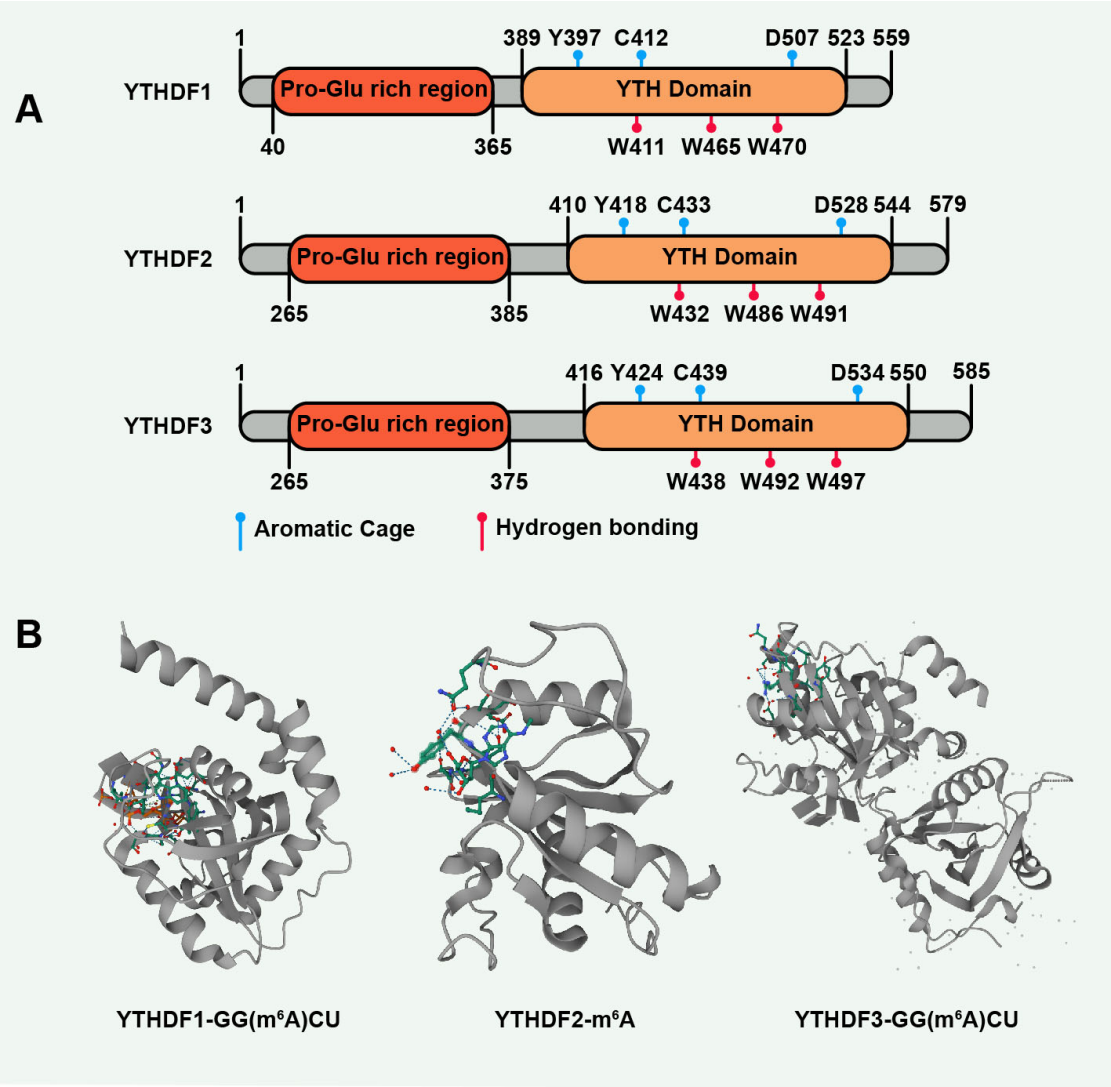
The structures of the YTHDF family, especially the YTH domain. **(A)** The YTH domain of YTHDFs: YTHDF1 (UniProt ID: Q9BYJ9), YTHDF2 (UniProt ID: Q9Y5A9), YTHDF3 (UniProt ID: Q7Z739). **(B)** Structures of YTHDFs in complex with m6A. YTHDF1 (PDB ID:4RCJ), YTHDF2 (PDB ID:4RDN), YTHDF3 (PDB ID:6ZOT). The secondary structures of proteins are shown in gray, and RNA molecules are shown in color.

The crystal structures of the three YTH domains and their complexes with an m^6^A mononucleotide (or m^6^A oligoribonucleotides) have been revealed (45, 46). The YTH domains share a mixed α-helix-β-sheet fold, where the α-helices surround a barrel-shaped center arranged by the β-sheets. The surface of the YTH domain has a positively-charged groove in which m^6^A is tightly locked. Specifically, m^6^A is located in a hydrophobic pocket formed by three highly conserved aromatic residues called an aromatic cage. In the YTHDF-m^6^A complex, the m^6^A adenine moiety is sandwiched between the rings of two aromatic residues, paralleling them (Trp411 and Trp470 in YTHDF1, Trp432, and Trp491 in YTHDF2, Trp438, and Trp497 in YTHDF3). And the methyl group of m^6^A points to the ring of one aromatic residue (Trp465 in YTHDF1, Trp486 in YTHDF2, Trp492 in YTHDF3) (36, 47, 48). As well as aromatic residues, some amino acids (aa) of the YTH domain also interact with m^6^A. For example, the backbone NH of Tyr397 in YTHDF1 and Tyr418 in YTHDF2 form hydrogen bonds with the N3 of m^6^A. The carbonyl oxygen of Cys412 in YTHDF1, Cys433 in YTHDF2, and Cys439 in YTHDF3 bind to the N6 of m^6^A by hydrogen bonding. To sum up, the Π-Π interactions between the m^6^A adenine moiety and the aromatic cage, the cation-Π interactions between the methyl group and the aromatic cage, and a series of hydrogen bonds lay a foundation for m^6^A recognition (36) (**Figure 2**).

Evidence confirms that the YTHDF family plays an integral role in the translation and degradation of m^6^A-modified mRNAs. YTHDF2 is the most explored YTHDFs and is generally expressed at much higher levels than YTHDF1 and YTHDF3 in most cells (42). YTHDF2 binds to m^6^A-modified mRNAs and recruits the CCR4-NOT deadenylase complex through its N-terminal 101-200 aa to initiate deadenylation, which is a prior condition of P-body localization and decay of targeted mRNAs (10, 49, 50). Additionally, m^6^A-modified mRNAs can also bind to YTHDF2 in an HRSP12-dependent manner, and subsequently cleaved by RNase P/MRP (endoribonucleases) (51, 52). In particular, HRSP12 bridges the N-terminal 100 aa of YTHDF2 and RNase P/MRP, contributing to the rapid degradation of mRNAs. And m^6^A-containing circular RNAs (circRNAs) are also degraded by this pathway. Interestingly, under heat shock stress, nuclear-translocated YTHDF2 protects m^6^A motifs in the 5’ untranslated region (5’UTR) of stress-induced transcripts and activates cap-independent translation initiation (53). The N-terminal of YTHDF1 (100-200 aa) is in charge of the translation of mRNAs with m^6^A modifications (54). YTHDF1 not only transports more mRNAs to translation machinery and promotes ribosome occupancy, but also enhances the translation-initiation rate by correlating eIF4G-mediated loop structure through interaction with eIF3 in a cap-dependent manner (17). YTHDF1 can also trigger translational elongation through interaction with elongation factors in some cancer cells (55–57). Apart from the above results, Li et al. found that YTHDF1 interacts with Argonaute 2 (AGO2) to stimulate the production of P-bodies for mRNA degradation (58). In addition, YTHDF3 augments m^6^A-mRNA translation by cooperating with YTHDF1 and interacting with the 40s/60s ribosome subunits (59). Besides that, YTHDF3 recruits eIF4G2 to m^6^A sites, driving translation initiation of circRNAs (60). YTHDF3 also promotes m^6^A-modified mRNA decay by working together with YTHDF2 (37). A recent study found that the effect of YTHDF3 in regulating targeted mRNA deadenylation during somatic cell reprogramming relies on the recruitment of the PAN2-PAN3 deadenylase complex (61).

Interestingly, the YTHDF family forms a classic functional model: upon entry into the cytoplasm, m^6^A-modified mRNAs are first bound by the YTHDF3 or YTHDF3-YTHDF1 complex and then recognized by YTHDF2, thereby regulating the different fates of the targeted mRNA (62). Nevertheless, it has recently been discovered that YTHDFs have redundant functions to a large extent (63). Those three YTHDFs share highly homologous structures (about 85% of aa sequence similarity) (64), similar RNA-binding properties (20), and a similar set of binding proteins, jointly regulating mRNA destiny in an m^6^A-dependent manner (65). Indeed, the distinct functions of YTHDFs depend on their expression levels, spatial locations, and post-translational modifications. Also, YTHDFs are affected by additional RNA-binding proteins that interact with YTHDFs, such as fragile X mental retardation protein (FMRP) (66, 67), and Proline-rich coiled-coil 2 A (Prrc2a) (68). Collectively, the role of YTHDFs in regulating gene expression is complex and requires further investigation.

## 4 The roles of the YTHDF family in physiological and pathological processes

### 4.1 Embryonic development

Among the three YTHDFs, YTHDF2 is expressed and plays a pivotal role throughout mammalian gametogenesis. YTHDF2-knockout female mice are infertile while male mice are hypo fertile (65, 69). Specifically, YTHDF2 is intrinsically required for oocyte competence to support early zygotic development rather than MII oocytes formation and fertilization process (69). YTHDF2 regulates appropriate maternal transcript dosage during oocyte maturation by selectively mediating transcript destabilization. Additionally, YTHDF2 clears m^6^A-dependent matrix metallopeptidase transcripts to promote the adhesion and proliferation of spermatogonia during spermatogenesis (70). Knockout of YTHDF2 results in morphologically deformed and functionally impaired sperm, even severe loss (65, 71).

Intriguingly, unlike the previous view that maternal mRNAs clearance and maternal-to-zygotic transition (MZT) are dependent on YTHDF2, Kontur et al. found that individual YTHDFs deletion does not prevent embryonic development, whereas double mutations of YTHDF2/YTHDF3 disrupts oogenesis and triple YTHDF depletion causes lethality in zebrafish (72, 73). Despite evidence for the redundant functions of YTHDFs in early mouse embryonic development, depletion of YTHDF2 causes lethality at late embryonic development stages with embryos exhibiting severe neurological deficits (65, 74). Zheng et al. found that YTHDF3 reduction is an adaptive mechanism under a hypoxic environment in early embryonic development (75). Specifically, YTHDF3 binds to the m^1^A site of insulin-like growth factor 1 receptor (IGF1R) mRNA and degrades IGF1R mRNA, hindering migration and invasion of trophoblast.

### 4.2 Stem cell fate

Somatic cells are reprogrammed into induced pluripotent stem cells (iPSCs), which have unlimited proliferation and pluripotent differentiation potential similar to human embryonic stem cells (ESCs) (76). YTHDF2 and YTHDF3 play an essential role in this reprogramming process by clearing somatic mRNAs, especially Tead2, through distinct m^6^A-dependent deadenylation mechanisms (61). While YTHDF1 is capable of increasing the expression of the transcription factor Btg2 and promoting the reprogramming of induced neuronal cells (77). In terms of iPSCs functions, the YTHDF1/YTHDF2 orchestration is involved in METTL3-m^6^A-mediated maintenance of pluripotent state in porcine iPSCs by elevating JAK2 level, reducing SOSC3 expression, and provoking STAT3/KLF4/SOX2 signal axis (78). YTHDF1 upregulation depends on MATR3 and maintains a MATR3-mediated pluripotent state in human iPSCs by maintaining the expression of OCT4 and LIN28A transcripts (79). Importantly, YTHDF2 is overexpressed and disrupts the expression of a group of m^6^A-modified mRNAs associated with neurodevelopment, thereby blocking neural differentiation and promoting pluripotency in human iPSCs (80). Similarly, YTHDF3 reduces gene expression associated with the formation of three germ layers, and the absence of YTHDF3 impairs pluripotency in ESCs (81).

Several studies have revealed that the specification and characteristics of hematopoietic stem cells (HSCs) are significantly regulated by YTHDF2. The m^6^A-YTHDF2-mediated decay of Notch1 mRNA is critical for the generation of the earliest hematopoietic stem/progenitor cells (HSPCs) during the endothelial-to-hematopoietic transition (EHT) in both zebrafish and mice embryos (82, 83). Li et al. first reported that YTHDF2 specifically mediates the ex vivo expansion of human HSCs due to the regulation of the stability of multiple mRNAs essential for HSC self-renewal (84). Therefore, inhibition of YTHDF2 makes it possible to obtain a sufficient number of HSCs from human umbilical cord blood (hUCB), which facilitates the application of hUCB HSCs transplantation. Furthermore, YTHDF2 deletion also promotes the expansion and regeneration of HSCs by eliminating the decay of both WNT-targeted and survival-related genes under stress conditions (85). Interestingly, although YTHDF2 is dispensable for steady-state multilineage hematopoiesis, long-term deficiency of YTHDF2 dramatically impairs HSCs activity and blocks reconstitution of multilineage hematopoiesis (86). Given that hematopoietic-specific YTHDF2 deficiency-induced long-term HSCs impairment is consistent with the adverse consequences of inflammation in HSCs, the inflammation-induced increase in YTHDF2 may be a protective mechanism for the long-term integrity of HSCs. YTHDF3 is also involved in the regulation of HSCs. YTHDF3 binds m^6^A on the 5’UTR of CCND1 mRNA and cooperates with PABPC1 and EIF4G2 to promote the expression of CCND1, a positive regulator of HSCs reconstitution capacity (87). While YTHDF3 facilitates the translation of FOXM1 and ASXL1 transcripts and is critical for maintaining HSC properties under stress conditions (88).

YTHDF1 is indispensable for maintaining intestinal stem cells (ISCs) during regeneration after intestinal damage by driving a positive feedback loop of the YTHDF1/TCF4/WNT signaling axis (89). Similarly, YTHDF1 sustains the stemness of ISCs through a targeted translation of transcriptional-enhanced associate domain 1 (TEAD1) (90). In addition, YTHDF1 is also involved in the m^6^A-mediated self-renewal of mouse female germline stem cells (mFGSCs) (91).

### 4.3 Fat metabolism

YTHDFs play key roles in adipogenesis, particularly YTHDF2. YTHDF2 binds and degrades JAK1 mRNA to block the JAK1/STAT5/C/EBPβ pathway, thereby inhibiting the adipogenic differentiation of bone marrow stem cells (92). Similarly, YTHDF2-mediated silencing of the JAK2/STAT3/C/EBPβ pathway impedes adipogenesis (93). Indeed, YTHDF2 also impairs adipogenesis by degrading multiple target transcripts through methylation-dependent modifications. Cell cycle factors, including CCNA2, CDK2, and CCND1 promote cell cycle progression and mitotic clonal expression in adipocytes (94, 95). Epigallocatechin gallate (EGCG) and metformin reduce CCNA2 and CDK2 levels by increasing m^6^A modification in an FTO-YTHDF2-dependent manner (96, 97). Conversely, Zinc finger protein (Zfp217) binds and sequesters YTHDF2 to reduce m^6^A levels, thus reversing CCND1 mRNA degradation (98). YTHDF2 also reduces the content of FAM134B, fatty acids synthesis-related proteins such as FASN, and autophagy-related proteins, including ATG5 and ATG7, which inhibit adipogenesis (99–101). Furthermore, the liver Bmal1 regulates the circadian clock of lipid metabolism by controlling the abundance of m^6^A modifications on transcripts (102). Mechanistically, Bmal1 knockdown inhibits PPARα expression in an m^6^A-YTHDF2-dependent manner, which increases lipid accumulation. Moreover, AMPK upregulates CD36 levels through YTHDF2-dependent Parkin reduction, which enhances intestinal long-chain fatty acid uptake and induces obesity in high-fat diet mice (103).

Intriguingly, YTHDF1 inhibits ovine adipogenesis and promotes porcine adipogenesis by promoting the expression of PNPLA2 and MTCH2, respectively (62, 104). Chen et al. found that YTHDF1 restrains PPARγ expression in mice by promoting the translation of m^6^A-modified TRAF4 transcripts, while curcumin exerts an anti-obesity role by reducing the effect of ALKBH5 demethylation on TRAF4 m^6^A modification (105). In addition, YTHDF1 together with METTL3 amplifies the function of Rubicon that inhibits autophagy by stabilizing Rubicon mRNA, and further blocks the clearance of lipid droplets (LDs) in mouse nonalcoholic fatty liver disease (NAFLD) (106).

### 4.4 Neuromodulation

YTHDF1 mainly regulates axonal function as well as learning and memory, and YTHDF2 is mainly involved in neural development and differentiation. Functional axon regeneration under peripheral nervous system injury is supported by m^6^A-YTHDF1-derived increases in global protein translation (107). And YTHDF1 is a key player in enhancing Robo3.1 mRNA translation and guidance of pre-crossing commissural axons in the spinal cord, whereas YTHDF1 is inhibited by floor plate-induced signals in post-crossing axons guidance (108). Furthermore, dual depletion of YTHDF1/YTHDF3 affects spine morphology and excitatory synaptic transmission in hippocampal neurons (109). Further study revealed that YTHDF1 accelerates basal transmission and long-term potentiation of synapses by advancing neuronal stimulation-induced protein translation, thereby promoting learning and memory, especially long-term memory (110). In a Drosophila short-term memory experiment, memory-storing neurons require YTHDF to maintain normal memory function during aging (111). Furthermore, YTHDF1-mediated Dvl1 mRNA translation has a synergistic effect with YTHDF2-mediated Wnt5a mRNA degradation in inhibiting axon growth of cerebellar neurons (112).

During neural development, YTHDF2 is overexpressed and positively regulates early brain development by promoting the proliferation and differentiation of neural stem/progenitor cells (NSPCs) (74). Knockout of YTHDF2 significantly reduces cerebral cortical thickness and induces differentiated neurons to produce abnormal stress-sensitive neurites. Interestingly, YTHDF2-silenced NSPCs cannot differentiate into glial cells. Wu et al. showed that YTHDF2 competes with Prrc2a for binding to Olig2 mRNA, resulting in impaired oligodendrocyte specification and myelination (68). Moreover, YTHDF2 is detrimental to the extension and maintenance of retinal ganglion cell (RGC) dendritic arborization (113).

YTHDFs are also involved in a variety of brain disorders. For example, downregulated miR-421-3p in microglia after cerebral artery occlusion/reperfusion (MCAO/R) relieves the repression of YTHDF1, thereby promoting p65 mRNA translation, leading to aggravated inflammation and brain injury (114). Impairments of fine motor and cognitive function in young mice exposed to multiple sevoflurane are attributable to a specific decrease in YTHDF1 expression (115). Overexpression of YTHDF1 ameliorates diabetes-induced cognitive impairment (116). Additionally, elevated YTHDF2 under persistent light impedes cognitive behavior in mice by perturbing the stability of TrkappaB mRNA (117). And a recent case report found that most individuals with YTHDF3 haploinsufficiency show intellectual disability and/or developmental delay of variable degrees (118).

### 4.5 Cardiovascular effect

YTHDF1 promotes cardiomyocyte (CM) differentiation, whereas YTHDF3 does the opposite (81). YTHDF1, which is positively regulated by ALKBH5, also promotes CM proliferation in injury-induced cardiac regeneration by enhancing YAP mRNA translation (119). Xu et al. indicated that YTHDF2 degrades Myh7 mRNA to mitigate cardiac hypertrophy during heart failure development (120). Conversely, lncRNA MIAT-induced YTHDF2 high expression stimulates cardiac hypertrophy by downregulating CPT-1a levels in the PPARα pathway (121). Moreover, YTHDF1 and YTHDF2 promote ocular pathological angiogenesis *via* the METTL3-m6A-LRP6 axis and the FTO-m6A-FAK axis, respectively (122, 123). YTHDF1/YTHDF2 cooperation stimulates the atherogenic inflammatory cascade in the vascular endothelium by upregulating NLRP1 and downregulating KLF4 (124). Furthermore, loss of either YTHDF1 or YTHDF2 alleviates the proliferation of pulmonary arterial smooth muscle cells and pulmonary hypertension under hypoxia. Mechanistically, YTHDF1 promotes the translation of MAGED1 mRNA while YTHDF2 activates the PI3K/AKT signaling pathway by degrading PTEN mRNA (125, 126). And YTHDF3 knockout protects lung epithelial cells from inflammatory injury by inhibiting inflammatory cytokine secretion after hypoxia/reoxygenation (127).

### 4.6 Viral infection

YTHDFs play anti-viral roles in the life cycle of Epstein-Barr virus (EBV), Hepatitis B virus (HBV), Hepatitis C virus (HCV), Zika virus (ZIKV), and enterovirus 71 (EV71) (128–133). For example, the knockdown of each DF in EBV-infected cells promotes EBV lytic replication and reactivation. Mechanistically, YTHDF1 attracts ZAP, DDX17, and DCP2 forming RNA degradation complexes to accelerate the decapping of m^6^A-modified RNAs and degrade EBV cleavage gene transcripts (128). Furthermore, activation of caspases cleaves D166 and D367 sites on YTHDF2 upon EBV reactivation reduces YTHDF2 expression, thereby increasing caspase-8 protein levels and enhancing EBV replication (129). Alternatively, YTHDFs inhibit HCV infection by reducing viral particle production rather than blocking viral RNA replication (131). During the chronic HCV infection state, YTHDFs relocate to lipid droplets, bind to the m^6^A site in the HCV E1 region, and antagonize viral packaging caused by the binding of the viral core protein to the non-m^6^A site in the E1 region. In contrast, YTHDF2 promotes simian virus 40 (SV40) and influenza A virus (IAV) replication (134, 135). Moreover, YTHDF1 and YTHDF3 induce severe acute respiratory syndrome coronavirus 2 (SARS-CoV-2) infection, YTHDF1 inhibits chikungunya virus (CHIKV) infection, and YTHDF2 functions opposite to that of YTHDF1 in both SARS-CoV-2 and CHIKV (136–138).

Notably, the regulation of YTHDFs in the transcription and replication of human immunodeficiency virus type 1 (HIV-1) and Kaposi’s sarcoma-associated herpesvirus (KSHV) remains controversial. Evidence suggests that YTHDFs hinder HIV-1 replication in target cells contradicting previous views that YTHDFs increase viral transcript and protein levels (139–141). Specifically, after HIV-1 infection into cells, YTHDFs impede HIV-1 reverse transcriptase by degrading incoming HIV-1 genomic RNA (gRNA) in an m^6^A-dependent manner, thereby limiting viral replication (139). Nevertheless, YTHDFs facilitate HIV-1 structural protein Gag synthesis and virus release, while forming a complex with HIV-1 Gag protein and viral and cellular RNAs in virus-producing cells (140). To ensure optimal HIV-1 infectivity, HIV-1 protease cleaves YTHDF3, which enters the virion in a nucleocapsid-dependent fashion (142). Additionally, Hesser et al. showed that YTHDF2 exerts pro- and anti-KSHV effects in iSLK and B cell lines, respectively (143). Instead, Tan et al. observed that YTHDF2 inhibits KSHV gene expression and virion production in iSLK cells (144). Together, the paradoxical phenomenon of YTHDFs in viral regulation may be explained by differences in cell types, viral life cycle stages, and experimental approaches.

### 4.7 Immunity

The type I interferon (IFN) signaling pathway relies on the expression of IFN-stimulated genes (ISGs) to mediate a powerful innate antiviral immune response. YTHDF1-mediated upregulation of IFITM1, a subset of ISGs, initiates antiviral responses (145). Another study showed that YTHDF1 prevents viral double-stranded RNA (dsRNA)-driven IFN responses (146). YTHDF1 induces the IFN-mediated expression of ADAR1, which disrupts the secondary structure of dsRNA in an adenosine-to-inosine (A-to-I) RNA editing manner. Furthermore, YTHDF2 deletion enables increased levels of IFN-βand inflammatory factors, including interleukin-6 (IL-6) by stabilizing host antiviral transcripts (147, 148). YTHDF2 also binds and sequesters m^6^A-modified viral RNA, which protects viral RNA from RIG-I recognition, thereby inhibiting RIG-I activation and the downstream IFN signaling pathway (149, 150). In contrast, YTHDF2 is an essential cofactor for the IFN-α-induced degradation of m^6^A-methylated HBV RNA by ISG20 (151). Additionally, enterovirus 2A proteases cleave YTHDFs and limit antiviral responses during early viral infection (152). Among them, the cleavage of YTHDF3 dampens the IFN-I-stimulated JAK/STAT signaling pathway. Interestingly, only YTHDF3 attenuated ISGs expression in the absence of viral infection (153). Mechanistically, YTHDF3 rapidly translates forkhead box protein O3 (FOXO3) mRNA through cooperation with PABP1 and eIF4G2 in an m^6^A-independent way, thereby suppressing ISGs expression.

Inflammatory responses are also an important part of immunity. YTHDF1 counteracts the excessive and persistent development of inflammation in the septic response by promoting the expression of SOCS1, a negative regulator of macrophage-mediated inflammation (154). However, YTHDF1 knockout suppressed inflammatory lung or intestinal damage (155, 156). Macrophage-specific YTHDF1 knockdown may be a protective therapy against brain injury in severe sepsis rats with ECMO by enhancing adaptive immune function and alleviating inflammatory damage (157). YTHDF2 also negatively regulates inflammation. YTHDF2 inhibits the MAPK and NF-κB signaling pathways by downregulating the expression of MAP2K4, MAP4K4, STAT1, and PPAR-γ, and subsequently prevents macrophage polarization and proinflammatory cytokine secretion (158–160). And YTHDF2-dependent decay of KDM6B mRNA restricts H3K27me3 demethylation, which impedes transcription of proinflammatory cytokine genes (161).

**Figure 3:**
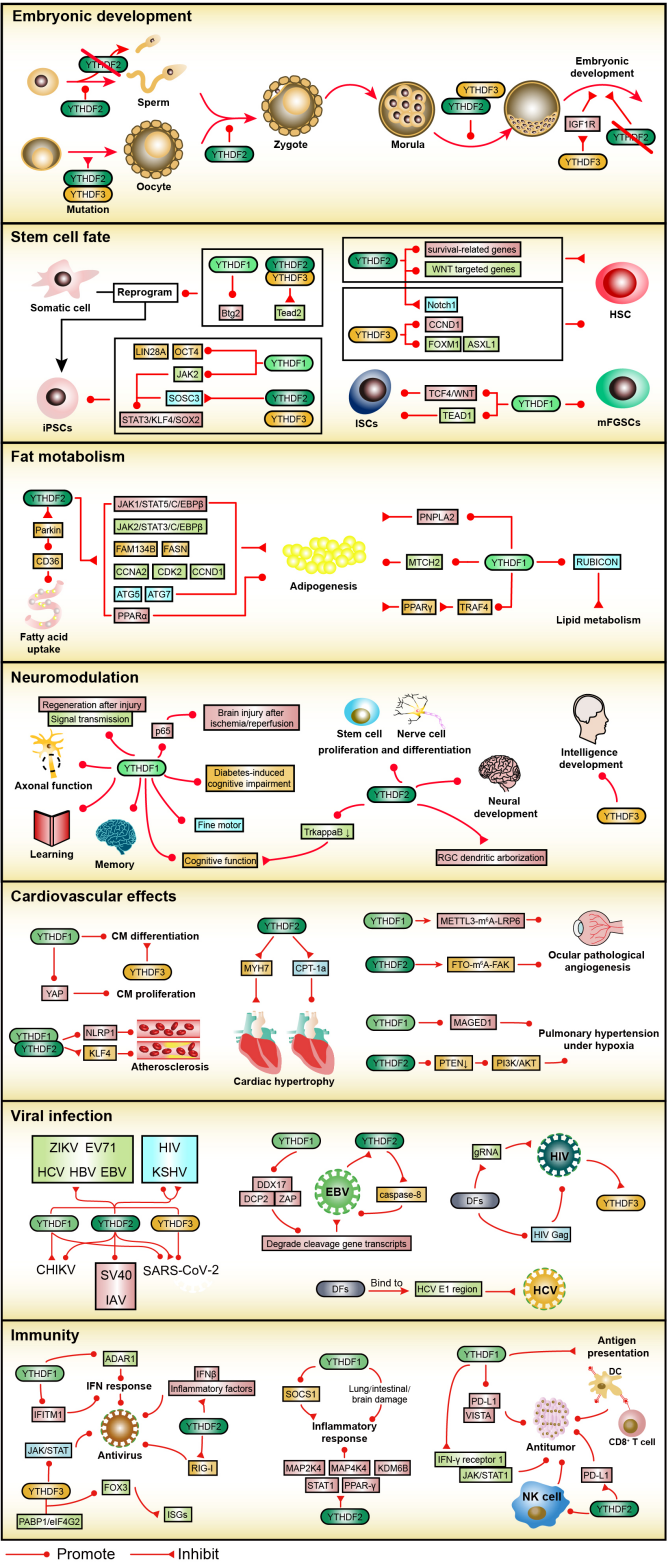
The roles of the YTHDF family in embryonic development, stem cell fate, fat metabolism, neuromodulation, cardiovascular effect, viral infection, and immunity. In embryonic development, YTHDF2 is essential for sperm, oocyte, zygote, and embryo formation. In stem cell fate, the YTHDF family promotes somatic cell reprogramming and the properties of iPSCs. In addition, YTHDF2 and YTHDF3 participate in the fate of HSC, and YTHDF1 in the fate of ISCs as well as mFGSCs. In fat metabolism, YTHDF1 and YTHDF2 regulate adipogenesis and fatty acid metabolism. In neuromodulation, YTHDF1 affects axonal function as well as learning and memory, YTHDF2 regulates neural development and differentiation, and YTHDF3 participates in intellectual development. In cardiovascular effect, YTHDF1 and YTHDF2 are closely related to the fate of CM, vascular endothelial cells, and pulmonary artery smooth muscle cells. In a viral infection, the YTHDF family is involved in the life cycle of several viruses, especially EBV, HCV, and HIV. In immunity, the YTHDF family plays an important role in antiviral immunity, inflammatory immunity, and anti-tumor immunity.

Strikingly, the expression of YTHDFs has a strong relationship with the immune regulation of various tumors. The expression of YTHDF1 is not only the highest in normal immune cells but also dramatically correlated with tumor immune-infiltrated cells in cancer, especially CD8^+^ T cells, macrophages, and dendritic cells (DCs) (162). Han et al. revealed that YTHDF1 is an important target for anti-tumor immunotherapy (163). YTHDF1 depletion accelerates tumor antigen presentation and cross-priming of CD8^+^ T cells by retarding lysosomal cathepsin translation in DCs in an m^6^A-dependent manner. And the loss of YTHDF1 recruits DCs and activates IFN-γ receptor 1 and JAK/STAT1 signaling pathways, thereby promoting antitumor immunity in GC (164). Li et al. demonstrated that YTHDF1 hinders CD8^+^ T cell infiltration and increases immune checkpoint expression, such as PD-L1 and V-domain Ig suppressor of T cell activation (VISTA), in CRC (165). To this end, YTHDF1 consumption can be synergistic with anti-PD-1/PD-L1 immunotherapy for effective anti-tumor therapy. Similarly, YTHDF2-deficient tumors increased the sensitivity to anti-PD-1/PD-L1 immunotherapy by stabilizing PD-L1 mRNA in ICC (166). However, YTHDF2 participates in anti-tumor and anti-viral infection by regulating the maturation, proliferation, and effector functions of NK cells (167) (**Figure 3**).

## 5 The role of the YTHDF family in cancers

### 5.1 Digestive system cancers

#### 5.1.1 Liver cancer

Studies have reported that YTHDF1 is an oncogene that is highly expressed and positively correlates with the pathology stage in hepatocellular carcinoma (HCC) (168, 169). YTHDF1 is also an independent factor for an unfavorable HCC prognosis. Lin et al. suggested that Snail induces epithelial-mesenchymal transition (EMT) to enhance the metastasis of HCC cells. Mechanistically, m^6^A-modified CDS facilitates translational elongation of the Snail mRNA in a YTHDF1/eEF2-dependent manner (55). In addition, the YTHDF1-mediated aggressive phenotypes are also associated with the activation of the AKT/GSK-3β/β-catenin pathway (170). Chi et al. perceived that the effect of YTHDF1 in enhancing HCC proliferation can be antagonized by hsa-miR-139-5p (171). YTHDF1 also promotes HCC cell growth by upregulating the PI3K/AKT/mTOR signaling pathway (172). Hu et al. showed that METTL3-m^6^A-YTHDF1-mediated RBM14 overexpression promotes Kupffer cell polarization and HCC progression (173). Furthermore, YTHDF1 is involved in the regulation of HCC under hypoxic stress. For example, hypoxia-inducible factor-1α (HIF-1α)-mediated upregulation of YTHDF1 promotes autophagy-associated genes ATG2A and ATG14 translation, thus aggravating HCC malignancy behavior (174). FOXO3 is a negative regulator of hypoxia-induced autophagy and mediates the sorafenib sensitivity in HCC (175). Importantly, YTHDF1 binds to METTL3-methylated m^6^A modification in the FOXO3 mRNA 3’UTR and increases its mRNA stability rather than translation. Moreover, under the sublethal heat stress from insufficient radiofrequency ablation (IRFA), YTHDF1 binds to the m^6^A site on the 5’UTR of EGFR mRNA and triggers EGFR translation, eventually resulting in HCC recurrence after IRFA (176).

Notably, YTHDF3 is also reported as a potential oncogene in HCC. YTHDF3 enhances HCC metastasis by maintaining ZEB1 mRNA stability in an m^6^A-dependent mechanism (177). YTHDF3/integrin subunit alpha 6 (ITGA6) is positively regulated by the lysine-specific demethylase 5B (KDM5B)/microRNA-448 axis and thereby enhances the self-renewal of HCC cells (178).

Intriguingly, YTHDF2 has a paradoxical effect on HCC in different studies. Zhong et al. professed that hypoxia-induced YTHDF2 downregulation reverses the repression of YTHDF2 on the ERK/MAPK signaling pathway, subsequently removing the inhibitory effect of YTHDF2 on the proliferation and growth of HCC cells (179). Mechanistically, YTHDF2 suppresses the activation of the ERK/MAPK signaling pathway by selectively recognizing the m^6^A site at the 3’UTR and triggering EGFR mRNA degradation. Hou et al. confirmed that YTHDF2 is significantly downregulated in HCC cells and YTHDF2 deficiency elicits inflammation, vascular abnormalization, and metastatic progression (180). Specifically, YTHDF2 destabilizes the mRNA of m^6^A-modified interleukin 11 (IL11) and serpin family E member 2 (SERPINE2) to exert an inhibitory effect. Conversely, YTHDF2 is also considered a tumor-promoting factor in HCC (181, 182). Yang et al. discovered that microRNA-145 targets the 3’UTR of YTHDF2 mRNA to attenuate its expression and thereby inhibits the proliferation of HCC cells (183). And YTHDF2 participates in METTL3-m^6^A-mediated HCC malignancy by shortening the half-life of the suppressor of cytokine signaling 2 (SOCS2) mRNA (184). Additionally, YTHDF2 increases the m^6^A levels in the 5’UTR of OCT4 mRNA in tandem with promoting OCT4 expression, eventually accelerating the HCC cancer stem cell (CSC) phenotype and metastasis (185). And PA2G4 depends on YTHDF2 to stabilize FYN mRNA and promote EMT-induced HCC metastasis (186). The discrepancy in the effect of YTHDF2 on HCC may be due to different cellular microenvironments or tumor heterogeneity (187).

In addition, YTHDF1 and YTHDF2 facilitate the advancement of intrahepatic cholangiocarcinoma (ICC) through increasing EGFR mRNA translation and IFIT2 mRNA decay, respectively (188, 189). Meanwhile, YTHDF2 silencing restrains ICC resistance to the exposure of cisplatin by reversing the degradation of cyclin-dependent kinase inhibitor 1B (CDKN1B) mRNA (190).

#### 5.1.2 Gastric cancer

YTHDF1 mutations occur in approximately 7% of gastric cancer (GC) patients, and high expression of YTHDF1 is correlated with high-risk progression and poor prognosis in patients (191–193). YTHDF1 deficiency is capable to attenuate GC progression, including proliferation and metastasis *in vitro* and *in vivo*. Mechanistically, YTHDF1 relies on m^6^A modification to promote the translation of frizzled7 (FZD7) and USP14, which transmit WNT/β-catenin signaling and AKT/ERK signaling, respectively (192, 193). In addition, METTL3 promotes the malignancy behavior of GC through YTHDF1/eIF3a-dependent post-transcriptional translation of SPHK2 (194).

Zhang et al. showed that the knockdown of YTHDF2 inhibits GC cell proliferation and accelerates apoptosis *in vitro* (195). And lncRNA LINC00470 relies on YTHDF2 to degrade m^6^A-containing PTEN mRNA and thus promote GC advancement (196). Additionally, the HIF-1α-induced increase of lncRNA-CBSLR suppresses ferroptosis and chem-sensitive under hypoxic stress through the YTHDF2-CBS-ACSL4 axis (197). Specifically, CBSLR contributes to CBS mRNA destabilization by binding to the m^6^A site on the CDS of CBS mRNA by recruiting YTHDF2. However, Shen et al. found that YTHDF2 plays a suppressive role in GC by destabilizing FOXC2 mRNA (198).

#### 5.1.3 Pancreas cancer

Among the YTHDF family, YTHDF2 is the most studied protein in pancreatic cancer. YTHDF2 is elevated in pancreatic cancer and orchestrates the migration/proliferation dichotomy (199). Specifically, YTHDF2 prevents EMT, migration, and invasion by downregulating YAP signaling and enhances proliferation by activating AKT/GSK3B/CCND1 pathway. However, YTHDF2 downregulates the levels of PERP and PER1 mRNA to promote cell proliferation and migration in an m^6^A-dependent manner (200, 201). METTL3-m^6^A-YTHDF2-mediated decay of nucleobindin 1 (NUCB1) mRNA counteracts the effects of NUCB1 in halting pancreatic cancer growth and augmenting the antitumor with gemcitabine (GEM) (202). Conversely, another study showed that the rs142933486 G>T polymorphism in PIK3CB improves PIK3CB mRNA and protein levels by derailing m^6^A-YTHDF2-dependent degradation mechanisms, which is significantly associated with the poor prognosis of PTEN-deficient pancreatic cancer patients (203). And compared with PIK3CB[T], YTHDF2 mainly binds to PIK3CB[G]. Similarly, FTO reverses YTHDF2-regulated degradation of platelet-derived growth factor C (PDGFC) mRNA and promotes cell proliferation by reactivating the AKT signaling pathway (204). Notably, YTHDF1 is associated with the immune microenvironment and prognosis of pancreatic cancer (205–207). A recent study found that a novel antineoplastic drug, Olean-28,13β-lactam (B28), inhibits glutamine metabolism by reducing the expression of YTHDF1, which induces pancreatic cancer cell death (208). In addition, YTHDF3-mediated downregulation of lncRNA DICER1-AS1 reverses the repression of glycolysis by miR-5586-5p in pancreatic cancer (209).

#### 5.1.4 Colorectal cancer

In colorectal cancer (CRC), YTHDF1 may be a molecular target for diagnosis and treatment (210). Mechanistically, elevated YTHDF1 in CRC is mainly attributed to an increase in DNA copy number (211). The oncogene c-MYC, WNT signaling, and APC mutation can also upregulate YTHDF1 expression at the translational level (89, 212). Further studies found that YTHDF1 promotes tumorigenicity and CSC-like activity by amplifying the WNT/β-catenin pathway with little effect on normal intestinal development (211). And deletion of YTHDF1 in ISCs shrinks tumor size and prolongs the lifespan of CRC-formed mice substantially. YTHDF1 can promote CRC progression and metastasis by translating m^6^A-modified Rho/Rac guanine nucleotide exchange factor 2 (ARHGEF2) mRNA and activating RhoA signaling (213). Furthermore, circular RNA protein tyrosine kinase 2 (circPTK2) restores the miR-136-5p-mediated repression of YTHDF1 by competitively binding to miR-136-5p, resulting in the CRC advancement and chemoresistance (214). Chen et al. suggested that YTHDF1-mediated glutamine metabolism reduces the sensitivity of CRC cells to cisplatin (215). Specifically, YTHDF1 targets the m^6^A of glutaminase 1 (GLS1) mRNA 3’UTR to promote its translation. And METTL3 deletion inhibits LDHA mRNA translation by reducing the binding of YTHDF1 to LDHA mRNA CDS, thereby hindering glycolysis and promoting 5-fluorouracil sensitivity in CRC cells (216). Interestingly, the rs8100241 G>A mutation in ANKLE1 increases ANKLE1 levels in an m^6^A-YTHDF1-dependent fashion, thereby inhibiting proliferation and maintaining the genomic stability of CRC (217).

In addition, YTHDF2 often collaborates with “writers” and participates in CRC progression. For example, METTL3 downregulates YPEL5 in an m^6^A-YTHDF2-dependent manner and boosts CRC progression (218). METTL14 exerts an inhibitory effect in CRC by promoting the degradation of SYR-related high-mobility-group box 4 (SOX4) mRNA and long noncoding RNA XIST, which is dependent on YTHDF2 (219, 220). Han et al. deciphered that glutaminolysis inhibition increases ATF4 expression through FTO-mediated demethylation and YTHDF2-regulated decay, which further inactivates mTOR and promotes pro-survival autophagy of CRC cells (221). Moreover, in CRC, silencing of microRNA-6125 destabilizes GSK3β mRNA by upregulating the expression of YTHDF2, ultimately increasing WNT/β-catenin/Cyclin D1 pathway-related proteins and promoting CRC growth (222). Intriguingly, Zhou et al. found that HIF-1α-induced upregulation of lncRNA STEAP3-AS1 activates the WNT/β-catenin signaling pathway through overexpression of STEAP3, leading to CRC progression in a hypoxic environment (223). Specifically, after combining YTHDF2, STEAP3-AS1 prohibits STEAP3 mRNA from binding with YTHDF2, thus antagonizing STEAP3 mRNA decay.

Moreover, Ni et al. revealed that the long noncoding RNA GAS5-YAP-YTHDF3 axis forms a feedback loop in CRC (224). In detail, the downregulation of GAS5 enhances CRC proliferation and invasion by inhibiting phosphorylation and ubiquitin-mediated decay of YAP, which positively regulates YTHDF3. And YTHDF3 promotes the degradation of GAS5 mRNA by recognizing the m^6^A in GAS5 mRNA. Furthermore, YTHDF3 recruits eIF2AK2 and eIF3A on the 5’UTR of target mRNAs and promotes translation in oxaliplatin-resistant CRC (225).

### 5.2 Respiratory system cancers

The expression of YTHDF1 and YTHDF2 is markedly upregulated in tumor tissues of lung cancer series and possesses tumor-promoting activities (226). Shi et al. demonstrated that YTHDF1 is amplified and increases the translation of key regulators of the G0/G1 cell cycle transition, including CDK2, CDK4, and cyclin D1 mRNAs, intensifying non-small cell lung cancer (NSCLC) progression under normoxia conditions (227). In addition, microRNA-376c, delivered by endothelial cells through extracellular vesicles, inhibits the YTHDF1 and WNT/β-catenin pathway in NSCLC cells, resulting in the malignant progression of NSCLC cells (228). Nevertheless, under cisplatin-induced oxidative stress, YTHDF1 deficiency activates the antioxidant Nrf2-AKR1C1 axis by inhibiting the Keap1 mRNA transition, which resulted in cisplatin resistance and poor prognosis. Furthermore, the YTHDF1-m^6^A-enolase1 (ENO1) translation axis is a crucial pathway for stimulating glycolysis and tumorigenesis (229). In KRAS and TP53 co-mutated lung adenocarcinomas, YTHDF1 recognizes m^6^A modification and contributes to tumor proliferation and poor prognosis through the upregulation of cyclin B1 (230).

In addition, YTHDF2 promotes translation but not clearance of 6-phosphogluconate dehydrogenase (6PGD) mRNA in an m^6^A-dependent manner by interacting with eIF3a/b, which enhances the pentose phosphate pathway (PPP) flux for tumor growth (231). The transcriptional repressor ZBTB4 and the tumor suppressor DAPK2 are negatively regulated by YTHDF2 and significantly associates with smoking-induced lung cancer (232, 233). However, ALKBH5 attenuates YTHDF2-mediated downregulation of oncogenic drivers such as SOX2, SMAD7, and MYC, contributing to the progression of aggressive lung cancer with KRAS mutation/LKB1 loss (234). Furthermore, YTHDF2 produces a positive effect on lung adenocarcinoma progression through the mRNA decay of AXIN1, a negative regulator of the WNT/β-catenin pathway (235). YTHDF2 produces the same effect in a VIRMA-m^6^A-dependent fashion in lung adenocarcinoma and NSCLC by reducing BTG2 mRNA and DAPK3 mRNA stability, respectively (236, 237). Nevertheless, YTHDF2 induces sensitivity of lung adenocarcinoma to gefitinib *via* cleavage of circASK1 (238). Interestingly, YTHDF2 promotes proliferation and downregulates the FAM83D-TGFβ1-SMAD2/3 pathway to inhibit migration and invasion in lung adenocarcinoma cells (239). In lung squamous cell carcinoma, up-regulation of YTHDF2 under hypoxic conditions activates the mTOR/AKT signaling pathway and induces EMT to play a tumor-promoting role (240).

Interestingly, YTHDF1 and YTHDF2 regulate YAP expression by competitively binding to YTHDF3-m^6^A-YAP mRNA, thereby aggravating and attenuating the malignancy behavior of NSCLC, respectively (241). YTHDF1/3 recruits eIF3a/b to promote YAP mRNA translation, while YTHDF2/3 recruits AGO2 to promote YAP mRNA decay. And YTHDF3 indirectly increased YAP levels to empower NSCLC progression and drug resistance by enhancing MALAT1 mRNA stability (242).

### 5.3 Urogenital system cancers

#### 5.3.1 Bladder cancer

YTHDF family plays a tumor-promoting role in bladder cancer. Specifically, METTL3 and YTHDF1 are closely related to malignant transformation and tumorigenesis in the presence of chemical carcinogens, with the m^6^A-methylated 3’UTR promoting oncogene CDCP1 translation (243). Moreover, YTHDF1/3 promotes aggressive phenotypes by translating ITGA6 mRNA, while YTHDF2 facilitates migration by degrading the mRNAs of the tumor suppressors SETD7 and KLF4 (244, 245).

#### 5.3.2 Prostate cancer

YTHDF2 acts as a facilitator and is negatively regulated by miR-493-3p in prostate cancer (PCa) (246). Du et al. considered that KDM5A abrogates the inhibition of miR-495 on YTHDF2, and then upregulated YTHDF2 intensifies PCa progression by inducing m^6^A-MOB3B mRNA decay (247). In addition, YTHDF2 clears METTL3-mediated m^6^A-dependent mRNA of LHPP, NKX3-1, and USP4 (248, 249). The decrease of LHPP and NKX3-1 causes PCa proliferation and migration by inducing AKT phosphorylation. And downregulated USP4 promotes ARHGDIA expression by reducing ELAVL1 protein, thus accelerating invasion and metastasis of PCa. METTL14-mediated m^6^A modification of Thrombospondin 1 (THBS1) mRNA promotes PCa proliferation in a YTHDF2-dependent manner of transcriptome degradation (250).

#### 5.3.3 Breast cancer

In breast cancer, high expression of YTHDF1 and YTHDF3 is associated with gene copy number amplification and induces a poor prognosis (251, 252). YTHDF1 targets FOXM1 mRNA and positively regulates breast cancer progression (253). Additionally, hypoxia-mediated downregulation of miR-16-5p restored YTHDF1 expression, thereby promoting tumor glycolysis by enhancing PKM2 mRNA translation (254). Sun et al. demonstrated that YTHDF1 stabilizes E2F8 mRNA, which accelerates DNA damage repair and chemoresistance to adriamycin, cisplatin, and the PARP inhibitor olaparib in breast cancer cells (255). YTHDF1/eEF1-mediated translational elongation of KRT7 mRNA and YTHDF3-induced mRNAs translation of ST6GALNAC5, GJA1, and EGFR is involved in breast cancer lung and brain metastasis, respectively (57, 256). And YTHDF3 can be antagonized by miR-106b-5p (257). Moreover, YTHDF3 stabilizes ZEB1 mRNA to promote the invasion and migration of triple-negative breast cancer (TNBC) cells (258). Furthermore, YTHDF2 is upregulated in TNBC cells and prevents cell apoptosis (259, 260). YTHDF2 also targets the m^6^A site 5’UTR region of ATF3 mRNA to mitigate the resistance of breast cancer cells to tamoxifen (261).

#### 5.3.4 Ovarian cancer

YTHDF1 and YTHDF2 are considered oncogenes in ovarian cancer. YTHDF1 is recruited to the m^6^A site of EIF3C mRNA and stimulates EIF3C as well as overall protein translation (262). YTHDF1 also confers cisplatin-resistant ovarian cancer cells with CSC-like traits by promoting m^6^A-TRIM29 mRNA translation (263). Furthermore, FBW7 abrogates the mRNA degradation of YTHDF2 on pro-apoptotic gene BMF by inducing YTHDF2 decay, disrupting ovarian cancer progression (264). Moreover, YTHDF2 can be directly targeted and inhibited by miR-145 in ovarian cancer cells (265).

#### 5.3.5 Cervical cancer

In cervical cancer (CC) cells, YTHDF1 accelerates m^6^A-augmented glycolysis and cancer progression by promoting translational elongation of pyruvate dehydrogenase kinase 4 (PDK4) mRNA and stabilization of hexokinase 2 (HK2) mRNA (56, 266). Specifically, the YTHDF1/eEF-2 complex binds the m^6^A site of PDK4 mRNA at the 5’UTR and YTHDF1 recognizes the m^6^A-modified 3’UTR of HK2 mRNA. Furthermore, YTHDF1 plays a tumor-promoting role by facilitating mitosis-associated RANBP2 mRNA translation in an m^6^A-mediated approach, while YTHDF2 exerts the same role by degrading the tumor suppressor GAS5 mRNA (267, 268). YTHDF2 deficiency suppresses the proliferation of CC cells, promotes apoptosis, and arrests the cells at the S phase (269). YTHDF2 can also facilitate EMT and cisplatin resistance in CC cells by stabilizing AXIN1 mRNA (270).

#### 5.3.6 Endometrial cancer

YTHDF1 and YTHDF2 modulate the negative regulator PHLPP2 and positive regulator mTORC2 of AKT respectively, which is unfavorable to the tumorigenicity of the AKT pathway in endometrial cancer (EC) (271). In addition, YTHDF2-mediated transcript degradation of IRS1 is accompanied by inhibition of the AKT/MMP9 signaling pathway, thereby impairing the activity of endometrial cells (272). And YTHDF2 deficiency activates the WNT signaling pathway by reducing the decay of HOXB13 mRNA, and thus promotes EC invasion and metastasis (273). Conversely, YTHDF2 degrades lncRNA FENDRR to enhance the expression of SOX4, which ultimately promotes EC cell proliferation and hinders apoptosis (274).

### 5.4 Cancers in other systems

#### 5.4.1 Glioblastoma

YTHDF1 and YTHDF2 were found to be highly overexpressed in glioblastoma (GBM) tissues compared to normal tissues (275). YTHDF1 is required for maintaining GBM CSC properties and promoting proliferation, migration, and chemoresistance (276). And Musashi-1(MSI1) is a GBM hyper-oncogenic regulator and positively regulates YTHDF1 expression. YTHDF1 also assists METTL3 in increasing levels of ADAR1 and thereby stimulates GBM cell growth (277). In addition, YTHDF2 is positively regulated by the EGFR/SRC/ERK pathway and facilitates the malignancy progression of GBM by degrading downstream transcripts, including LXRα, HIVEP2, UBXN1, and ASS1 mRNAs in an m^6^A-dependent manner (43, 278, 279). Among them, LXRα and ASS1 are related to cholesterol homeostasis and arginine metabolism, respectively. Strikingly, YTHDF2 recognizes m^6^A methylation to maintain MYC mRNA stability, thereby promoting the expression of the downstream effector IGFBP3, leading to GBM CSC growth (280). And this process occurs specifically in GBM CSCs but not in normal neural stem cells (NSCs). Chen et al. verified that YTHDF2 promotes temozolomide desensitization in GBM cells (281). Mechanistically, YTHDF2 activates PI3K/AKT and NF-κB signaling pathways by targeting the 3’UTR and downregulating the mRNAs stability of EPHB3 and TNFAIP3.

#### 5.4.2 Melanoma

YTHDF1 is amplified in melanoma, and the combination of YTHDF1 and HNRNPA2B1 significantly increases the diagnostic validity (282). However, YTHDF1 inhibits ocular melanoma progression by facilitating HINT2 mRNA translation (283). YTHDF2 knockdown promotes tumor growth and reduces the sensitivity of anti-PD-1 therapy by enhancing the mRNAs stability of the intrinsic genes PD-1 (PDCD1), CXCR4, and SOX10 in an m^6^A-dependent fashion (284). Yu et al. discovered that histone lactylation promotes YTHDF2 expression in ocular melanoma, and YTHDF2 stimulates tumorigenesis by degrading m^6^A-modified PER1 and TP53 mRNAs (285). Similarly, YTHDF3 also promotes ocular melanoma progression by promoting CTNNB1 mRNA translation in an m^6^A-dependent manner (286).

#### 5.4.3 Merkel cell carcinoma

The occurrence of Merkel cell carcinoma (MCC) is mostly attributed to the attack of the small T antigen of Merkel cell polyomavirus (MCPyV) (287). Meanwhile, overexpression of YTHDF1 improves the proliferative and clonogenic capacity of MCC cells by recruiting eIF3a/b to promote the translation initiation of small T antigen mRNA. Mechanistically, overexpression of YTHDF1 is caused by increased gene copy number.

#### 5.4.4 Acute myeloid leukemia

**Figure 4:**
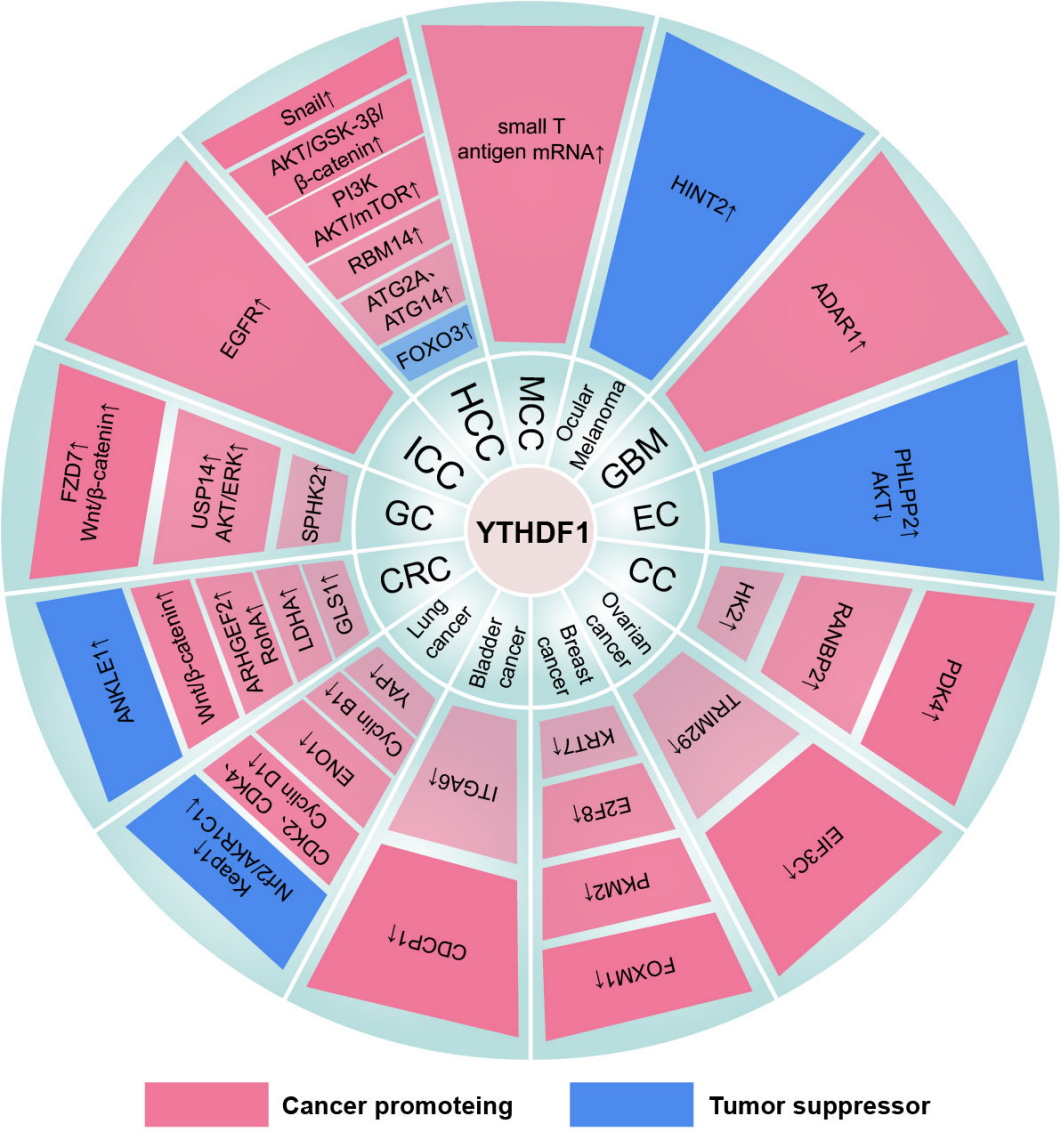
The mechanism of the YTHDF1 family in cancers. “↓” is the decrease of target mRNAs. “↑” is the increase of target mRNAs.

**Figure 5:**
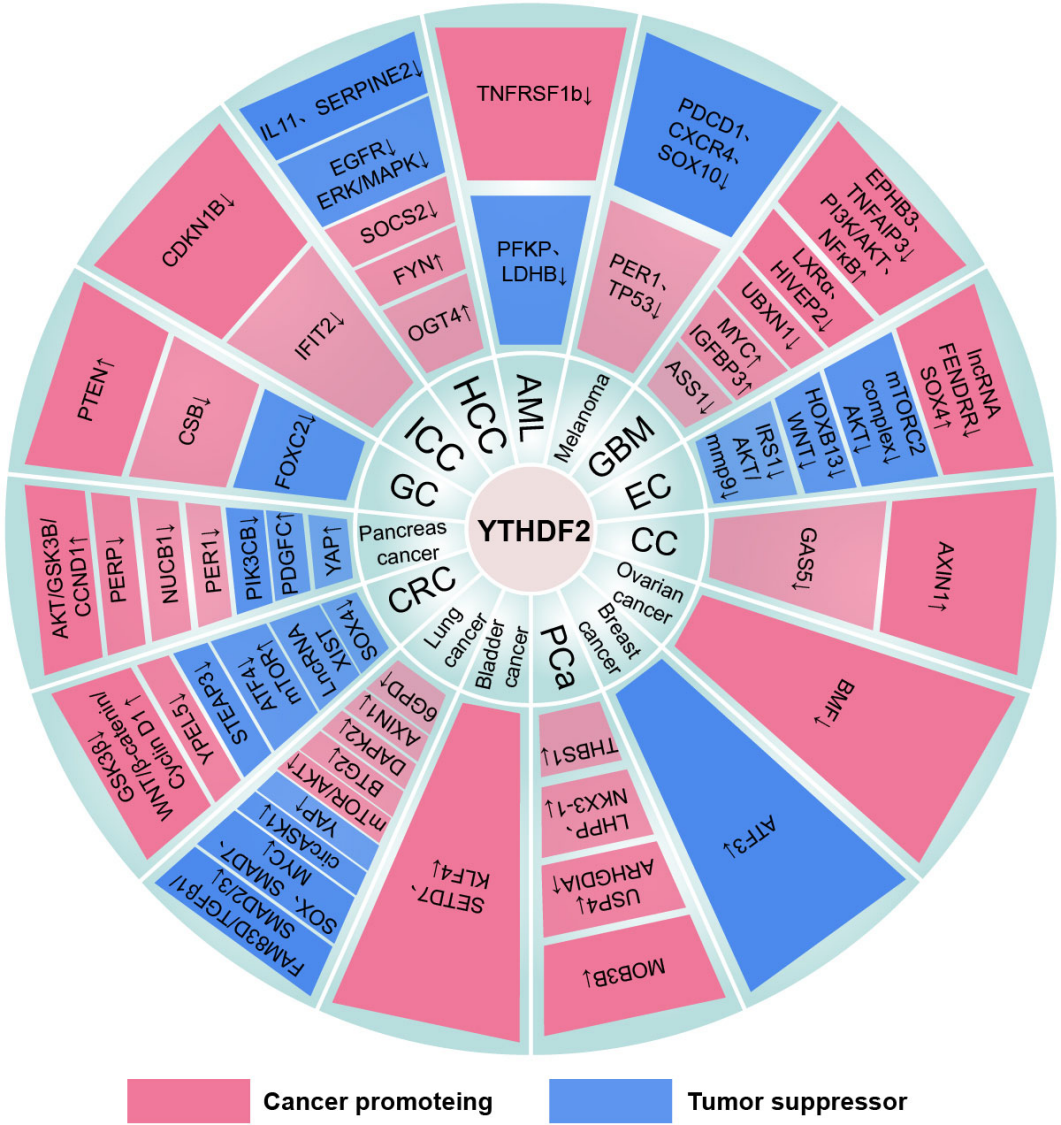
The mechanism of the YTHDF2 family in cancers. “↓” is the decrease of target mRNAs. “↑” is the increase of target mRNAs.

**Figure 6:**
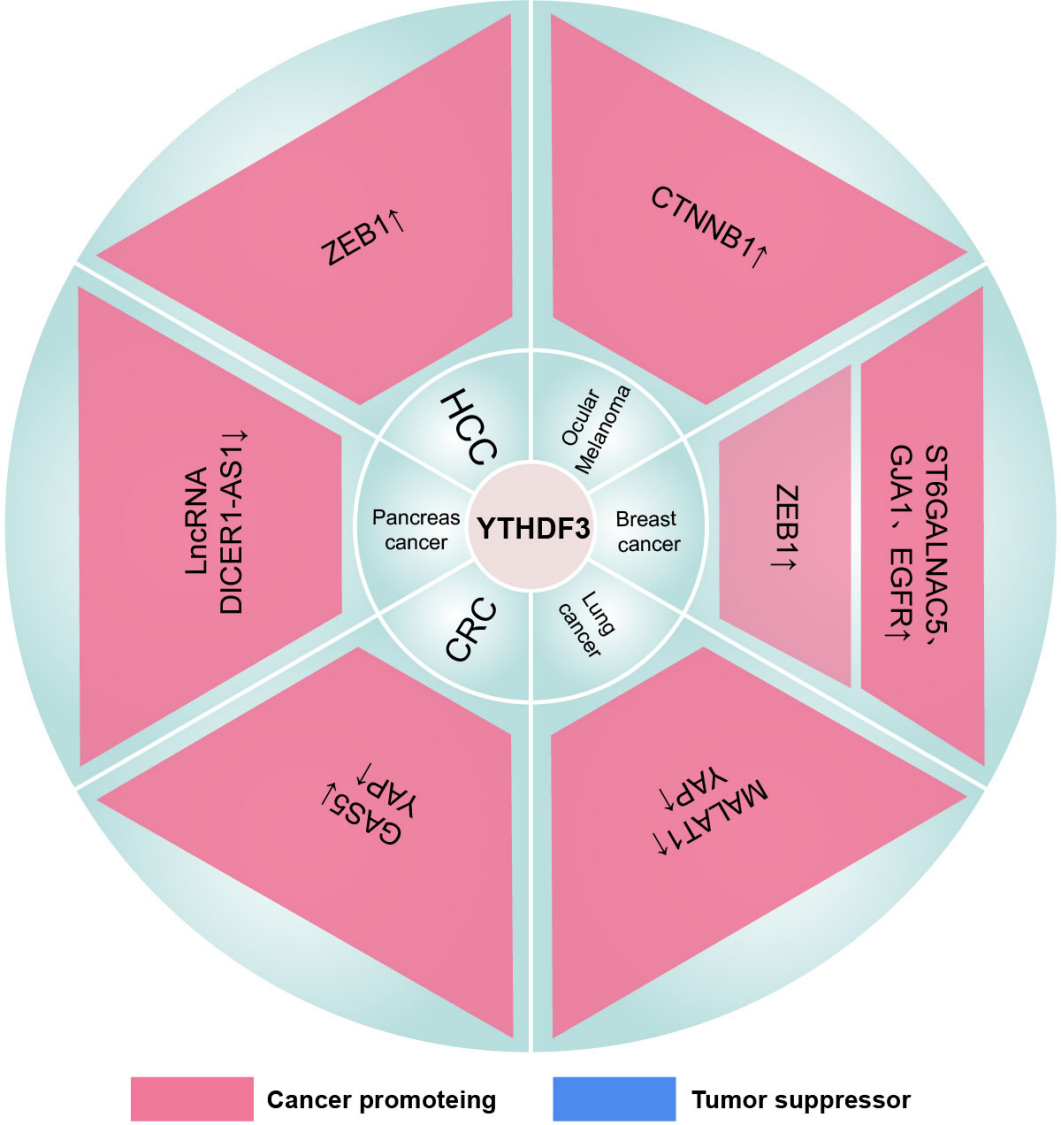
The mechanism of the YTHDF3 family in cancers. “↓” is the decrease of target mRNAs. “↑” is the increase of target mRNAs.

Nguyen et al. first reported that YTHDF2 is identified as a novel acute myeloid leukemia1 (AML1) T translocation partner gene (288). Notably, YTHDF2 is highly expressed in different AML subtypes (289). And inhibition of YTHDF2 specifically impairs AML initiation and progression while expanding hematopoietic stem cells (HSCs) and maintaining normal hematopoietic function. In detail, YTHDF2 promotes the development and propagation of AML CSCs by degrading multiple m^6^A-modified mRNAs such as TNF receptor superfamily member 1b (TNFRSF1b) that are associated with the functional integrity of AML CSCs. Moreover, the AML1/ETO-HIF1α loop transactivates the YTHDF2 promoter to promote t (8, 21) AML cell proliferation (290). However, YTHDF2 may interfere with the glycolytic process of AML cells by destabilizing transcripts of phosphofructokinase platelet (PFKP) and lactate dehydrogenase B (LDHB) (291). Interestingly, the three YTHDFs can jointly degrade the associated transcripts and inhibit the differentiation of AML cells (63) (**Figures 4**–**6**) (**Tables 1**–**3**).

**Table 1:** The role of the YTHDF1 in cancers.

Cancers	Roles	Cooperative m6A regulators	Target mRNAs	“Reading” position	The mechanism of target mRNAs	Functional classification	References
Hepatocellular carcinoma	Oncogene	METTL3	Snail	CDS	Promoting translation	EMT and metastasis	(55)
		METTL3	RBM14	–	Promoting expression	Growth and metastasis; Kupffer cells polarization	(173)
		–	ATG2A and ATG14	CDS	Promoting translation	Hypoxia-induced autophagy, growth, and metastasis	(174)
		METTL3	EGFR	5’UTR	Promoting translation	Viability and metastasis	(175)
	Tumor suppressor	METTL3	FOXO3	3’UTR	Increasing stability	Sorafenib sensitivity	(176)
Intrahepatic cholangiocarcinoma	Oncogene	–	EGFR	3’UTR	Promoting translation	Proliferation, migration, and invasion	(188)
Gastric cancer	Oncogene	–	FZD7	3’UTR	Promoting translation	Proliferation and metastasis	(192)
		–	USP14	CDS	Promoting translation	Proliferation and metastasis	(193)
		METTL3	SPHK2	–	Promoting translation	Proliferation, migration, and invasion	(194)
Colorectal cancer	Oncogene	–	ARHGEF2	3’UTR	Promoting translation	Growth and metastasis	(213)
		–	GLS1	3’UTR	Promoting translation	Cisplatin resistance	(215)
		METTL3	LDHA	CDS	Promoting translation	Glycolysis and 5-fluorouracil resistance	(216)
	Tumor suppressor	METTL3/14 and WTAP	ANKLE1	–	Promoting translation	Proliferation	(217)
Lung cancer	Oncogene	–	CDK2, CDK4, and cyclin D1	–	Promoting translation	Proliferation	(227)
		METTL3 and ALKBH5	ENO1	CDS	Promoting translation	Glycolysis and growth	(229)
		–	cyclin B1	3’UTR	Promoting translation	Proliferation	(230)
		METTL3 and ALKBH5	YAP	–	Promoting translation	Growth and metastasis	(241, 242)
	Tumor suppressor	–	Keap1	–	Promoting translation	Cisplatin sensitivity	(227)
Bladder cancer	Oncogene	METTL3 and ALKBH5	CDCP1	3’UTR	Promoting translation	Growth	(243)
		METTL3 and ALKBH5	ITGA6	3’UTR	Promoting translation	Adhesion, migration, and invasion	(244)
Breast cancer	Oncogene	–	FOXM1	CDS	Promoting translation	Proliferation and metastasis	(253)
		–	PKM2	CDS	Promoting translation	Glycolysis, growth, and metastasis	(254)
		METTL14	E2F8	–	Increasing stability	Growth, DNA damage repair, and chemoresistance	(255)
		FTO	KRT7	CDS	Promoting translation	Lung Metastasis	(57)
Ovarian cancer	Oncogene	–	EIF3C	–	Promoting translation	Proliferation and metastasis	(262)
		–	TRIM29	3’UTR	Promoting translation	The CSC-like phenotype	(263)
Cervical cancer	Oncogene	METTL3	PDK4	5’UTR	Promoting translation	Glycolysis, proliferation, and doxorubicin resistance	(56)
		METTL3	HK2	3’UTR	Increasing stability	Warburg effect and Proliferation	(266)
		–	RANBP2	–	Promoting translation	Growth, migration, invasion, and apoptosis	(267)
Endometrial cancer	Tumor suppressor	METTL3/14	PHLPP2	–	Promoting translation	Proliferation	(271)
Glioblastoma	Oncogene	METTL3	ADAR1	–	Promoting translation	Proliferation	(277)
Ocular melanoma	Tumor suppressor	METTL3 and ALKBH5	HINT2	3’UTR	Promoting translation	Growth and migration	(283)
Merkel cell carcinoma	Oncogene	–	small T antigen	–	Promoting translation	Proliferation and Cloning	(287)

The meaning of the symbol "-" is that the specific content has not yet been revealed in the corresponding research.

**Table 2:** The role of the YTHDF2 in cancers.

Cancers	Roles	Cooperative m6A regulators	Target mRNAs	“Reading” position	The mechanism of target mRNAs	Functional classification	References
Hepatocellular carcinoma	Oncogene	METTL3	SOCS2	–	Promoting degradation	Proliferation, migration, and colony formation	(184)
		–	OCT4	5’UTR	Promoting translation	CSC phenotype and cancer metastasis	(185)
		–	FYN	–	Increasing stability	EMT and metastasis	(186)
	Tumor suppressor	–	EGFR	3’UTR	Promoting degradation	Proliferation and growth	(179)
		–	IL11 and SERPINE2	3’UTR	Promoting degradation	Inflammation, vascular reconstruction, and metastatic progression	(180)
Intrahepatic cholangiocarcinoma	Oncogene	METTL3	IFIT2	–	Promoting degradation	Proliferation, apoptosis, cell cycle process, invasion, and migration	(189)
		METTL3	CDKN1B	–	Promoting degradation	Proliferation, apoptosis, cell cycle process, and cisplatin resistance	(190)
Gastric cancer	Oncogene	METTL3	PTEN	–	Promoting degradation	Proliferation, migration, and invasion	(196)
		METTL3	CBS	CDS	Decreasing stability	Ferroptosis and chemoresistance	(197)
	Tumor suppressor	–	FOXC2	–	Decreasing stability	Proliferation, migration, and invasion	(198)
Pancreas cancer	Oncogene	METTL14	PERP	3’UTR	Decreasing stability	Growth and metastasis	(200)
		ALKBH5	PER1	3’UTR	Promoting degradation	Proliferation and metastasis	(201)
		METTL3	NUCB1	5’UTR	Promoting degradation	Growth and GEM resistance	(202)
	Tumor suppressor	METTL3/14 and WTAP	PIK3CB	–	Decreasing stability	Proliferation and migration	(203)
		FTO	PDGFC	3’UTR	Decreasing stability	Proliferation	(204)
Colorectal cancer	Oncogene	METTL3	YPEL5	CDS	Promoting degradation	Growth and metastasis	(218)
		–	GSK3β	3’UTR	Promoting degradation	Proliferation	(222)
	Tumor suppressor	METTL14	SOX4	–	Promoting degradation	migration, invasion, and metastasis	(219)
		METTL14	XIST	–	Promoting degradation	Proliferation and metastasis	(220)
		FTO	ATF4	–	Decreasing stability	Autophagy	(221)
		METTL14	STEAP3	–	Promoting degradation	Proliferation and metastasis	(223)
Lung cancer	Oncogene	–	6PGD	3’UTR	Promoting translation	Growth	(231)
		METTL3	DAPK2	–	Decreasing stability	Proliferation and migration	(233)
		–	AXIN1	–	Promoting degradation	Proliferation and metastasis	(235)
		VIRMA	BTG2	3’UTR	Decreasing stability	Proliferation and metastasis	(236)
		VIRMA	DAPK3	3’UTR	Promoting degradation	Proliferation, migration, and invasion	(237)
	Tumor suppressor	ALKBH5	SOX2, SMAD7, and MYC	–	Decreasing stability	Proliferation and migration	(234)
		METTL3	circASK1	–	Promoting degradation	Gefitinib sensitivity	(238)
		–	FAM83D	–	Promoting degradation	Migration and invasion	(239)
		METTL3 and ALKBH5	YAP	–	Promoting degradation	Growth and metastasis	(241)
Bladder cancer	Oncogene	METTL3	SETD7 and KLF4	–	Promoting degradation	Migration	(245)
Prostate cancer	Oncogene	–	MOB3B	–	Promoting degradation	Proliferation, migration, invasion, and apoptosis	(247)
		METTL3	LHPP and NKX3-1	–	Promoting degradation	Proliferation and migration	(248)
		METTL3	USP4	CDS	Promoting degradation	Invasion and metastasis	(249)
		METTL14	THBS1	–	Promoting degradation	Proliferation	(250)
Breast cancer	Tumor suppressor	–	ATF3	5’UTR	Decreasing stability	Tamoxifen sensitivity	(261)
Ovarian cancer	Oncogene	–	BMF	3’UTR	Promoting degradation	Proliferation	(264)
Cervical cancer	Oncogene	ALKBH5	GAS5	–	Promoting degradation	Growth and metastasis	(268)
		–	AXIN1	–	Increasing stability	EMT and cisplatin resistance	(270)
Endometrial cancer	Oncogene	FTO	FENDRR	–	Promoting degradation	Proliferation and apoptosis	(274)
	Tumor suppressor	METTL3/14	mTORC2	–	Promoting degradation	Proliferation	(271)
		METTL14 and ALKBH5	IRS1	CDS	Promoting degradation	Proliferation and invasion	(272)
		FTO	HOXB13	3’UTR	Promoting degradation	Invasion and metastasis	(273)
Glioblastoma	Oncogene	–	LXRα and HIVEP2	–	Promoting degradation	Proliferation, invasion, and cholesterol dysregulation	(43)
		METTL3	UBXN1	–	Promoting degradation	Proliferation and migration	(278)
		METTL14	ASS1	–	Promoting degradation	Proliferation, migration, and invasion	(279)
		METTL3	MYC	–	Increasing stability	CSC growth	(280)
		–	EPHB3 and TNFAIP3	3’UTR	Decreasing stability	Temozolomide resistance	(281)
Melanoma	Tumor suppressor	FTO	PDCD1, CXCR4, and SOX10	5’UTR and 3’UTR	Promoting degradation	Growth and anti-PD-1 blockade immunotherapy sensitivity	(284)
Ocular melanoma	Oncogene	–	PER1 and TP53	3’UTR	Promoting degradation	Proliferation and migration	(285)
Acute myeloid leukemia	Oncogene	–	TNFRSF1b	–	Promoting degradation	The development and propagation of AML CSCs	(289)
		–	TNFRSF1b	3’UTR	Decreasing m^6^A levels	Proliferation	(290)
	Tumor suppressor	FTO	PFKP and LDHB	–	Promoting degradation	Glycolysis	(291)

The meaning of the symbol "-" is that the specific content has not yet been revealed in the corresponding research.

**Table 3:** The role of the YTHDF3 in cancers.

Cancers	Roles	Cooperative m6A regulators	Target mRNAs	“Reading” position	The mechanism of target mRNAs	Functional classification	References
Hepatocellular carcinoma	Oncogene	–	ZEB1	–	Increasing stability	Metastasis	(177)
Pancreas cancer	Oncogene	–	DICER1-AS1	–	Decreasing stability	Glycolysis, proliferation, and metastasis	(209)
Colorectal cancer	Oncogene	–	GAS5	–	Promoting degradation	Proliferation and invasion	(224)
Lung cancer	Oncogene	METTL3	MALAT1	–	Increasing stability	Cisplatin resistance, growth, and metastasis	(242)
Breast cancer	Oncogene	–	ST6GALNAC5, GJA1, and EGFR	–	Promoting translation	Brain metastasis	(256)
		–	ZEB1	–	Increasing stability	Migration, invasion, and EMT	(258)
Ocular melanoma	Oncogene	–	CTNNB1	–	Promoting translation	Proliferation and migration	(286)

The meaning of the symbol "-" is that the specific content has not yet been revealed in the corresponding research.

## 6 Limitations and perspectives

Although it has been revealed that the YTHDF family is involved in a variety of biological processes as the “readers” of m^6^A modification, there are still many mysteries about the YTHDF family that need to be discovered and solved in terms of structure, function, and treatment.

The discussion of the structure and function of YTHDFs is partially doubtful due to the limitations of technology and conditions. The reason why YTHDFs select the same or different target mRNAs and m^6^A sites on mRNAs, and why YTHDFs pair with different cooperating m^6^A regulators, has not been reached. In addition, YTHDFs can be localized in different cellular compartments and may re-enter the nucleus or transport out of the cell membrane, thus expanding the regulation of YTHDFs. The post-transcriptional modifications of YTHDFs and interactions of YTHDFs with other proteins also add to the structure and function complexity of YTHDFs. Therefore, the development of emerging technologies, the control of various conditions, and the change of different stimulus states are necessary to further investigations in the YTHDF family.

At present, many experiments have successfully constructed the YTHDF1/2/3 genetic KO mouse model using different techniques. First, the whole-body YTHDF1/2/3 KO mice are generated directly based on CRISPR/Cas9 by deleting a certain exon or inducing the premature appearance of a stop codon (87, 88, 110, 213). Second, the Cre/LoxP technique is used to generate cell-specific conditional YTHDF1/2/3 KO mice (74, 84, 108, 165, 167, 174, 227, 289). This represents an improvement in experimental research moving from *in vitro* to *in vivo*. However, the specific mutation of functional RNA binding sites of YTHDFs in mice needs to be further realized. In addition, one of the important purposes of experimental research is clinical transformation, so it is of great need to explore the application value of targeting YTHDFs in the clinic, especially in tumors. Many clinical-related studies have analyzed the expression profile of the m^6^A regulator in tumors and its association with the immune microenvironment, grading, staging, therapeutic effect, and prognosis. For example, the analysis of 162 HCC samples from the Zhou et al. and 177 HCC samples from the Nakagawa et al. showed that YTHDF1 was related to poor prognosis of HCC and YTHDF2 was related to HCC recurrence, respectively (169, 182). YTHDF1 was associated with a poor prognosis of GC in a study of 379 patients with GC (164). Interestingly, high expression of YTHDF1 and YTHDF2 was associated with a better prognosis in 603 cases of resected NSCLC, which might be due to increased tumor-infiltrating lymphocytes (TILs) and decreased co-inhibitor molecule PD-L1 (226). In addition, an assessment of single nucleotide polymorphisms (SNPs) in the YTHDF1 gene in 313 cases of hepatoblastoma showed that rs6090311 A>G was correlated with a reduced risk of hepatoblastoma (292). A similar SNPs assessment found that the YTHDF2 rs3738067 variant significantly increased glioma risk in 171 pediatric patients (293). Moreover, increasing evidence confirms the efficacy of bioinformatics analysis based on TCGA and other databases for the YTHDFs-associated model. To sum up, the expression of YTHDFs is significantly correlated with the grades and stages of various tumors and may be used as indicators to judge the occurrence and development of tumors. YTHDFs may act as independent prognostic factors for many tumors and affect survival-related indicators such as overall survival (OS), disease-free survival (DFS), and progression-free survival (PFS). At the therapeutic level, targeting YTHDFs can not only directly modulate the malignancy behavior of tumors, but also affect the sensitivity of chemotherapy and immunotherapy. Besides, YTHDFs also have the possibility of effective clinical application in non-cancer, including hematopoietic, anti-obesity, anti-viral, and anti-inflammatory.

**Figure 7:**
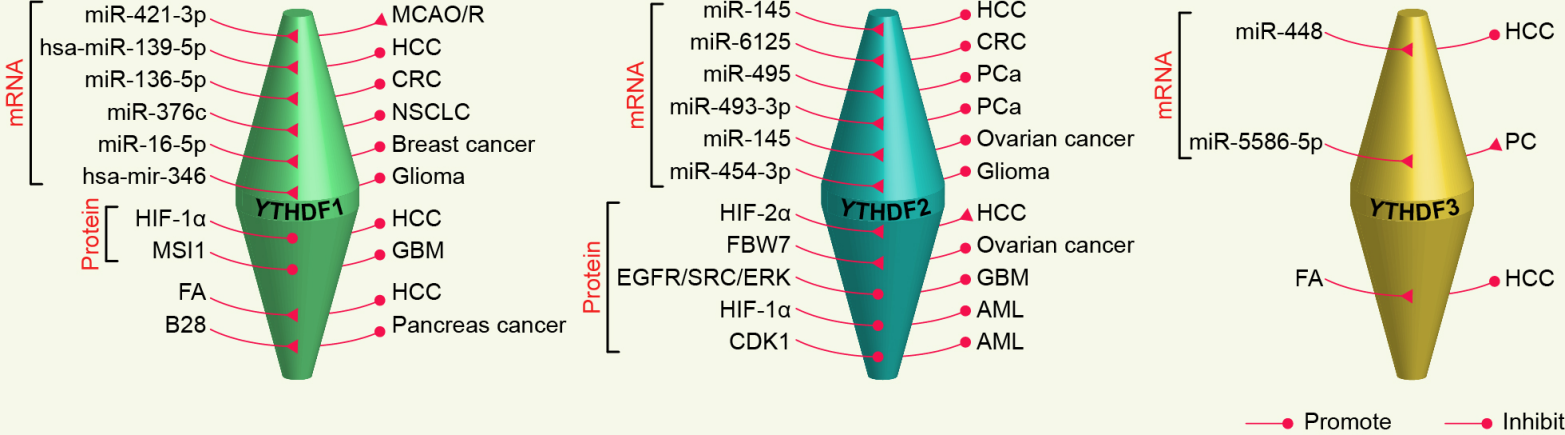
The upstream regulations of the YTHDF family.

However, studies of YTHDFs are still in the preclinical stage and many issues need attention. First, the clinical application of YTHDFs in different diseases, alone or in combination with other targets, requires further investigation. Second, the effectiveness of YTHDFs in diagnosing and predicting prognosis may vary across disease types, grades, and stages. Most importantly, the specific molecules targeting YTHDFs have not yet been developed. So how can YTHDFs be used in clinical treatment? The expression of YTHDFs can be regulated by other strategies. Targeting upstream or metabolic mechanisms of YTHDFs is an alternative approach to indirectly regulate the levels of YTHDFs (**Figure 7**). YTHDF2 has the capability of inhibiting the progression of HCC, and this effect can be antagonized by HIF-2α (180). Therefore, the HIF-2α antagonist (PT2385) can indirectly restore the effect of YTHDF2. And CDK1 inhibitors promote YTHDF2 proteolysis in AML (294). Furthermore, the delivery of target genes using viral vectors is also a feasible approach to target YTHDFs. YTHDF1 overexpression therapy can be achieved by injecting adeno-associated virus (AAV)-YTHDF1 into the hippocampus of diabetic cognitively impaired mice (116). In conclusion, clarifying the limitations of YTHDFs is conducive to better clinical transformation.

## 7 Conclusions

With multi-omics advancement, the roles of m^6^A modification have been gradually and seriously excavated. By binding to m^6^A, the YTHDF family plays an important role in the regulation of various physiological and pathological processes, including embryonic development, stem cell fate, fat metabolism, neuromodulation, cardiovascular effect, viral infection, immunity, and especially in tumors. In particular, YTHDFs regulate multiple tumor phenotypes such as proliferation, metastasis, metabolism, drug resistance, and immunity. Additionally, YTHDFs can be used as biomarkers for the diagnosis, treatment, and predictors of prognosis evaluation. On-going explorations of YTHDFs in modeling disease progression are still warranted for a better and deeper understanding of epigenetic modifications.

## Author contributions

LC collected the related papers and drafted the manuscript. YG made the figures and revised the manuscript. SX edited and revised the manuscript. JG designed the framework and revised the manuscript. JY, MW, and TL revised the manuscript. All authors contributed to the article and approved the submitted version.
